# Dynamic Boronate Ester Based Hydrogel with Enhanced Mechanical Properties and Multi-Stimuli-Triggered Release for Tissue Repair and Antioxidant Therapy

**DOI:** 10.3390/gels11050370

**Published:** 2025-05-18

**Authors:** Fangyi Liu, Gaoyang Li, Zhenhui An, Sijia Wang, Shouhong Xu, Honglai Liu

**Affiliations:** 1 Key Laboratory for Advanced Materials, School of Chemistry and Molecular Engineering, East China University of Science and Technology, Shanghai 200237, China; 13767201031@163.com (F.L.); 18021055632@163.com (G.L.); anzhenhui213@163.com (Z.A.); hlliu@ecust.edu.cn (H.L.); 2 College of Pharmacy, Henan University of Chinese Medicine, Zhengzhou 450046, China

**Keywords:** tissue repair, oxidative stress, controllable drug release, boronic ester bond, injectable hydrogel

## Abstract

Oxidative stress and chronic inflammation play pivotal roles in causing impaired tissue regeneration and delaying wound healing processes. Epigallocatechin gallate (EGCG) demonstrates robust anti-inflammatory and antioxidant characteristics, thereby emerging as a highly promising therapeutic substance for tissue repair applications. In order to counteract the pathological characteristics of the wound microenvironment, including increased levels of reactive oxygen species (ROS), low pH (weak acidic conditions), and elevated glucose concentrations, a hydrogel with pH/ROS/glucose-responsive properties was fabricated. This hydrogel was modified with phenylboronic acid (PBA) groups, which not only enhance its mechanical strength but also endow it with multi-stimuli responsiveness via dynamic boronate ester bonds. The impacts of grafting of PBA and loading of EGCG on the rheological and mechanical properties, as well as the network structure of the hydrogels, were systematically investigated. Moreover, in vitro experiments showed that the hydrogel could achieve excellent sustained and controlled release of both small-molecule and macromolecular drugs. Additionally, cell viability tests verified the hydrogel’s outstanding biocompatibility, and antioxidant experiments demonstrated its efficient ability to scavenge intracellular ROS. In conclusion, this injectable and biodegradable hydrogel possesses multi-stimuli responsiveness, controllable drug release behavior, and antioxidant capacity, presenting a promising approach to alleviate oxidative damage and promote tissue repair. This study offers valuable perspectives for the design of advanced hydrogel materials aimed at treating wound healing.

## 1. Introduction

In a normal tissue environment, the endogenous tissue system maintains a certain redox balance, which is beneficial for maintaining normal cell proliferation, differentiation, and the cascade reaction of apoptosis [1]. At the site of injury tissue, reactive oxygen species (ROS) are generated in the microenvironment to defend against invading pathogens [2,3]. However, the local accumulation of high levels of ROS and reactive nitrogen species (RNS), along with impaired free radical scavenging mechanisms, are the primary reasons for delayed wound healing. In tissue repair, especially in diabetic wound healing, local hyperglycemia at the lesion site and the resulting bacterial or fungal infections can disrupt the acid–base balance of the microenvironment. This imbalance leads to excessive accumulation of oxidative substances, often causing oxidative stress at the wound site. The high oxidative state of tissue cells stimulates signaling transcription, producing inflammatory factors that promote the generation of pro-inflammatory cells, triggering more severe inflammation [4]. Therefore, controlling oxidative stress and improving the inflammatory microenvironment are effective approaches to address delayed wound healing in tissue repair [5,6].

Bioactive substances [7] (such as antibiotics [8], anti-inflammatory drugs [9], exosomes [10], growth factors [11,12], etc.) can be used to promote wound tissue repair, but they have several disadvantages such as serious side effects, multidrug resistance, and easy deactivation [13]. For instance, EGCG, the most abundant polyphenol in green tea [11,14,15], has anti-inflammatory [16], antioxidant [17], antibacterial [18], anticancer [19], and angiogenesis-promoting [20] properties. It has been applied in various fields such as wound healing [21,22], cancer treatment [23,24], neuroinflammation therapy [25], and osteoarthritis repair [16]. However, EGCG has limitations of susceptibility to metabolism and inactivation, which necessitates the development of suitable drug delivery platforms to improve drug utilization effectively [26,27]. PBA-functionalized hydrogels not only protect the phenolic hydroxyl active site of EGCG through borate and hydrogen bonding, but also re-stabilize EGCG in normal physiological environments and achieve responsive release in damaged environments [28].

Hydrogels possess several properties, including large pore size, suitable mechanical strength, ease of functionalization, and high water retention capacity [24]. In the fields of tissue repair and regenerative medicine, natural biopolymers such as polysaccharides, proteins, and peptides, which exhibit excellent biocompatibility and biodegradability, are commonly utilized for the development of drug carriers and tissue engineering scaffold hydrogels [29]. Hydrogels for tissue repair are available in various forms, including dressings [30,31], microneedles [32], and foams [33,34]. Hydrogel-based drug delivery has been reported as a highly promising strategy due to its ability to significantly enhance therapeutic effects while minimizing systemic toxicity and drug wastage in bodily fluids, thereby improving overall treatment efficacy [35]. For instance, Sa Pang [36] et al. developed an oxidized hyaluronic acid hydrogel incorporating the metal–organic framework ZIF-8 as a pH-responsive drug delivery system for tendon adhesion treatment. Liang [37] et al. coordinately assembled the small-molecule drug rhein with anions (Ag^+^) to construct a dense, carrier-free hydrogel for sustained drug release, effectively treating bacterial infections while mitigating the risk of antibiotic resistance associated with traditional antimicrobial therapies. Additionally, Steverink [38] et al. synthesized a ring-shaped hydrogel containing bupivacaine crystals, reducing in vitro drug-induced cytotoxicity and enabling the stable release of non-opioid analgesics following spinal surgery.

**Figure 1:**
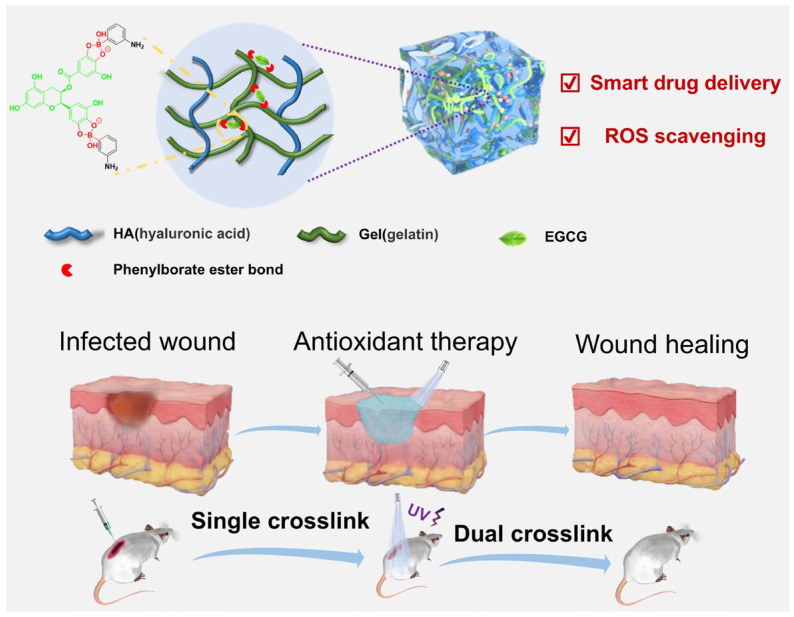
Schematic diagram of preparation of antioxidant hydrogel.

However, the weak mechanical strength of hydrogels often leads to irreversible damage, while their inherent network structure hinders effective encapsulation and controls the release of small-molecule drugs, ultimately limiting the effectiveness of treatment. In this study, we aimed to develop an injectable hydrogel drug delivery system with pH/ROS/glucose responsiveness for tissue repair. Boronic ester bonds, a typical reversible dynamic chemical bond [39], are formed by the coupling of boronic acid and diol structures, and they exhibit responsiveness to pH [28,40], ROS [41,42] and glucose [43,44]. The catechol groups in EGCG could form reversible boronic ester bonds with phenylboronic acid (PBA), constructing dynamic covalent bonds within the hydrogel that not only enhance the mechanical properties of the hydrogel but also enable controlled drug release. First, we modified natural polymers gelatin (Gel) and hyaluronic acid (HA) with methacrylic acid (MA) groups and modified GelMA with 3-aminophenylboronic acid (PBA). The catechol groups in EGCG formed boronic ester bonds with PBA, and hydrogen bonding occurred between the chains of HA and gelation, forming the single-crosslinked network. Subsequently, under ultraviolet (UV) irradiation, free radical polymerization of MA groups led to the formation of a dual-crosslinked network. The final hydrogel was an injectable hydrogel with multi-responsive antioxidant properties. The rheological and mechanical properties, as well as the biocompatibility of the hydrogel, were systematically investigated. Additionally, through the experiments of drug release and antioxidant capacity evaluation, the potential application of this drug-loaded hydrogel in promoting wound healing and antioxidant therapy has been verified, which is shown in Figure 1.

## 2. Results and Discussion

### 2.1. Design and Synthesis of the Hydrogels

**Figure 2:**
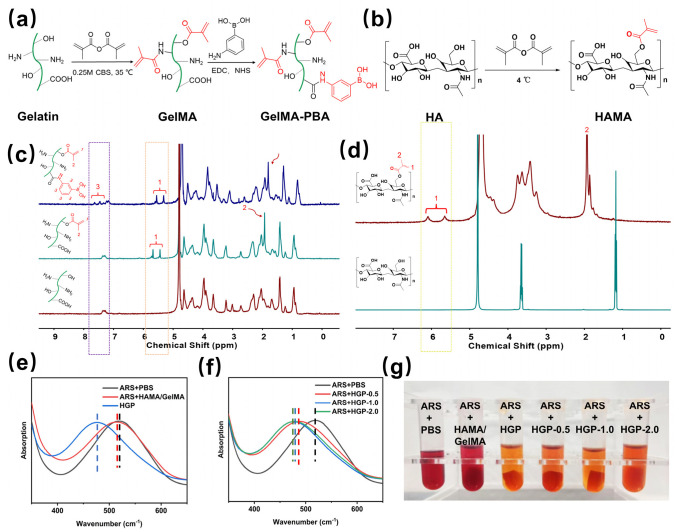
Preparation of GelMA-PBA, HAMA and hydrogel with various contents of EGCG. The synthesis scheme of (**a**) GelMA-PBA, (**b**) HAMA and the ^1^H NMR spectra of (**c**) GelMA-PBA, (**d**) HAMA, where red represents the position of the proton peak. UV adsorption of ARS solution added by (**e**) different hydrogels and (**f**) hydrogels with different concentrations of EGCG (**g**). Photographs of ARS solution added by different hydrogels.

Through the design of simultaneous grafting of MA and PBA onto the gelation side chains, PBA forms ester bonds with polyphenolic drugs, endowing hydrogels with controllable drug release behavior in response to stimuli. As shown in Figure 2a, the synthesis of GelMA-PBA was achieved by grafting MA onto gelatin through esterification, followed by an amide reaction to graft 3-aminophenylboronic acid, as demonstrated by the ^1^H NMR spectrum in Figure 2c. The double peaks at 5.6 and 5.8 ppm represent the vinyl group of GelMA, and the proton signal at 7.4–7.8 ppm corresponds to the phenyl group, confirming the successful modification of PBA on GelMA. Similarly, Figure 2b shows the synthesis route of HAMA. The degree of substitution (DS) of MA in gelation and HA was 76.2% and 64.7%, respectively, while the substitution degree of PBA on the GelMA side chain was 25.1%, determined by integrating the proton peaks at 5.7 and 6.2 ppm of ^1^H NMR spectra in Figure 2c,d.

After mixing and stirring GelMA-PBA, HAMA, and the small-molecule drug EGCG, the solution underwent free radical polymerization under UV irradiation to form a hydrogel. The formation of boronate ester bonds between PBA and EGCG was confirmed using ARS staining. As shown in Figure 2e–g, the grafting of PBA onto the HGP hydrogel resulted in a blue shift of the UV absorption peak, accompanied by a color change of the solution from red to yellow. Further introduction of EGCG induced a slight red shift in the UV absorption peak, with the solution color changing from yellow to a deep orange, which can be attributed to the binding of EGCG with PBA, leading to the release of ARS. FTIR spectroscopy further confirmed the successful modification of the polymers (see Figures S1 and S2 in the Supporting Information for details). For comparison, a control hydrogel without PBA, named HAMA/GelMA, was also prepared. The various hydrogels synthesized in this study are summarized in Table 1.

Finally, the obtained EGCG-carrying hydrogel possessed a double crosslinked network. The phenol hydroxyl group on EGCG first forms a borate ester bond with the PBA group on the gelatin side chain, and at the same time forms a hydrogen bond with amino groups and hydroxyl groups on the side chains of HAMA and GelMA, forming the single-crosslinked network. After UV irradiation, a new carbon-carbon single bond was formed between methacrylic acid groups due to a polymerization reaction, which formed a more stable dual-crosslinked network. The specific mechanism of EGCG cross-linking with gelatin and hyaluronic acid is illustrated in Figure S3 in the Supporting Information.

### 2.2. Morphology of Hydrogels

**Figure 3:**
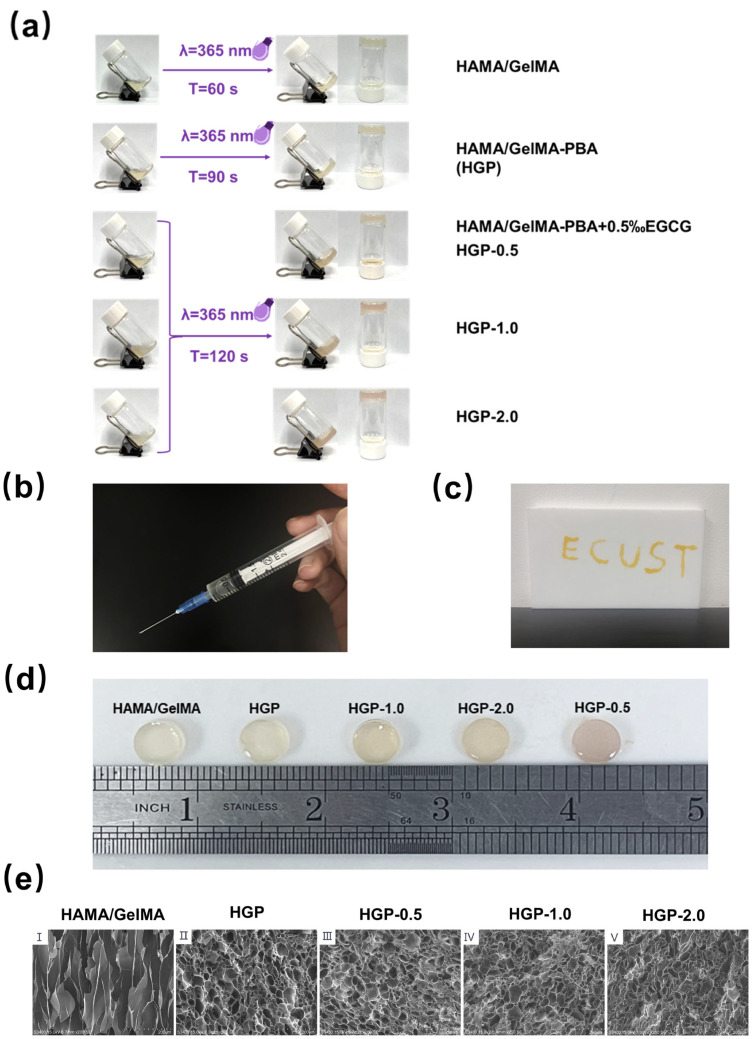
Morphology of hydrogels. (**a**) Time of hydrogel gelation after UV irradiation. (**b**) Injectability of hydrogel. (**c**) Shape adaptability. (**d**) Macromorphology of the hydrogel. (**e**) Micromorphology of hydrogels by SEM Scale bar: 200 μm.

The injectability of the hydrogels is crucial for treating irregular morphologies, and the gelation time also affects the practical application effect. As shown in Figure 3a, the gelation time of the hydrogels was observed to increase with the grafting of PBA and the loading of EGCG. Figure 3b,c show that the precursor solution of the hydrogel could be easily drawn into a syringe. After injection, it could form a hydrogel in situ under UV light, with good shape adaptability.

The macroscopic appearance of the hydrogels is shown in Figure 3d. As the EGCG content increased, the gel exhibited a slight brownish color, turning from transparent to semi-transparent. SEM images of the cross-section of the hydrogels (Figure 3e(I–V)) revealed that after the introduction of PBA, the hydrogel network became more prominent and well-rounded, indicating an increase in crosslinking. This is because after PBA grafting onto the gelation side chains, the hydrophobicity increased, and the hydrophobic interaction between PBA molecules facilitated crosslinking. As more EGCG was added, more multiple hydroxyl groups formed ester bonds with the PBA groups on the GelMA side chains, acting as network nodes. The increase in EGCG content reduced the pore size of the hydrogel network, indicating a further increase in crosslinking density. This conclusion is similar to the results of previous studies [45].

### 2.3. Rheological and Mechanical Properties of Hydrogels

**Figure 4:**
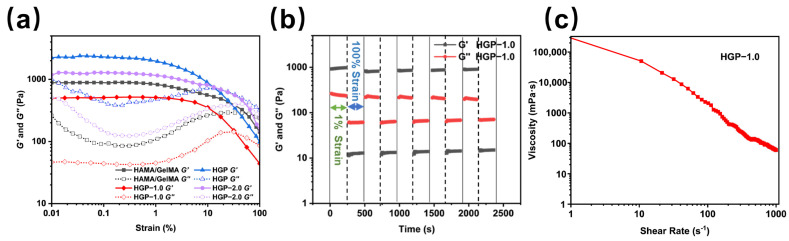
Characterization of physical properties of hydrogels. (**a**) Amplitude sweep measurement of various hydrogels. (**b**) Cyclic strain sweep and (**c**) viscosity measurement of HGP-1.0.

To investigate the rheological properties of the hydrogels, strain amplitude sweep tests were conducted to measure the storage modulus (G′) and loss modulus (G″), as shown in Figure 4a. For strains below 1%, both G′ and G″ remained nearly constant, with G′ significantly greater than G″, indicating the high stiffness and strong deformation resistance of the hydrogel. Between the 1% and 30% strain, G′ decreased sharply, suggesting that the hydrogel structure became unstable and began to deteriorate. When the strain exceeded 30%, G″ became greater than G′, indicating that the hydrogel structure collapsed and transitioned into a sol state. Comparison of the G′ values across different hydrogels revealed that the incorporation of PBA increased the G′ of the HGP hydrogel, indicating enhanced stiffness and reduced elasticity. However, with the addition of EGCG, G′ gradually decreased.

Figure 4b shows the cyclic strain test results of the HGP-1.0 hydrogel at 1% and 100% strain. Under high strain (100%, 250 s), G″ exceeded G′, leading to structural disruption of the hydrogel. When the strain was reduced to 1% (250 s), G′ became greater than G″, implying the sol reverted to gel form again. After five consecutive cycles of high and low strain, the hydrogel demonstrated 100% self-healing ability, whereas the HAMA/GelMA hydrogel exhibited no self-healing behavior. The self-healing capability of the hydrogel is attributed to the dynamic boronic ester bonds formed between PBA and EGCG, which enable the hydrogel to quickly restore its structure after damage. Figure 4c illustrates the viscosity of the HGP-1.0 hydrogel under varying shear forces, confirming its shear-thinning behavior.

**Figure 5:**
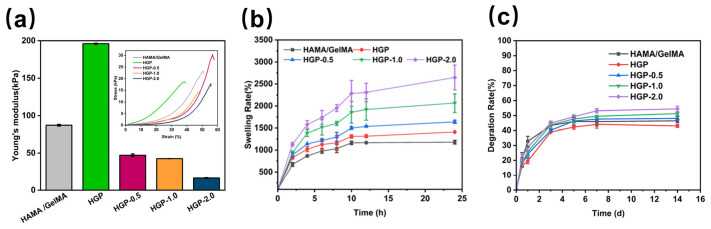
Characterization of physical properties of hydrogels. (**a**) Stress–strain curves (inset) and Young’s modulus, (**b**) swelling rate and (**c**) degradation rate of various hydrogels.

All hydrogels exhibited a certain degree of compressibility. As shown in the compression stress–strain behavior results in Figure 5a (Inset), which is consistent with storage modulus results, the HGP hydrogel had the lowest strain tolerance, approximately 37.8%. Young’s modulus calculated from Figure 5a further confirmed that the HGP hydrogel exhibited the highest modulus, indicating greater brittleness and rigidity. This is likely due to the introduction of PBA, which enhances hydrophobic interactions between GelMA chains, strengthening the primary crosslinked network and improving the mechanical strength of the hydrogel. In contrast, the HGP-2.0 hydrogel did not fracture until the strain exceeded 54.6%, suggesting that as the loading of the small-molecule drug EGCG increased, the proportion of hydrogen bonding increased, leading to a higher degree of crosslinking and a denser network. Consequently, the hydrogel exhibited reduced rigidity and enhanced flexibility. Meanwhile, Young’s modulus of our designed EGCG-loaded hydrogels (HGP-0.5, HGP-1.0, and HGP-2.0) was measured as (47.14 ± 1.79) kPa, (42.55 ± 0.13) kPa, and (16.29 ± 0.53) kPa, respectively. This trend may be attributed to changes in mechanical properties caused by the incorporation of EGCG. After crosslinking by EGCG, the hydrogel density increased, and its hardness improved accordingly. The results also demonstrate that the hydrogel’s properties can be effectively modulated by varying the EGCG content. Overall, Young’s modulus of the prepared hydrogels consistently falls within the range of 10–50 kPa, similar to that of natural skin tissue [46].

The water vapor transmission rate (WVTR) of the hydrogel is presented in Supporting Information Figure S4. The WVTR values were similar to those of healthy skin tissue (240–1920 g/m^2^) [47], indicating that the hydrogels can maintain breathability similar to normal skin tissue when applied to wound sites.

### 2.4. Swelling and Degradation Behavior of Hydrogels

The swelling capability of the hydrogels is essential for maintaining a moist environment, especially for epidermal tissue, to facilitate tissue repair. As shown in Figure 5b, the swelling ratio of the hydrogels indicated that EGCG-loaded hydrogels could absorb more than ten times their own weight in water. Among all drug-loaded hydrogels, HGP-2.0 (with 0.2% EGCG loading) had the highest swelling ratio, approximately (2647.3 ± 283.1) %, while HGP, HGP-0.5, and HGP-1.0 exhibited equilibrium swelling ratios of (1411.3 ± 6.3) %, (1638.5 ± 36.0) %, and (2067.3 ± 211.2) %, respectively. This suggests that the swelling ratio of the hydrogels increased with the incorporation of PBA and EGCG, likely due to the increased hydrogen bond content in the hydrogel structure, which enhanced its ability to bind water molecules. The degradation performance of the hydrogels, which is critical for medical applications, is shown in Figure 5c. The introduction of PBA reduced the degradation rate from (44.4 ± 1.8) % to (41.3 ± 1.3) %, and hydrogels with higher EGCG content showed a slightly increased degradation rate. However, the differences in degradation among these hydrogels were not significant.

### 2.5. In Vitro Drug Release Behavior of Drug-Loaded Hydrogels

**Figure 6:**
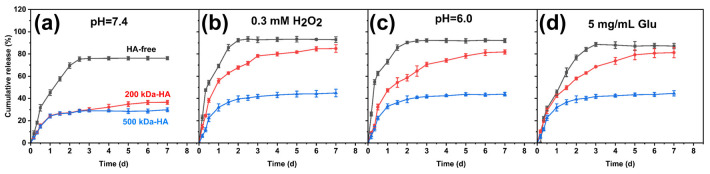
In vitro drug release profiles of EGCG-loaded hydrogels with different HA molecular weights under various conditions (black line: HA-free control; red line: 200 kDa-HA; blue line: 500 kDa-HA): (**a**) pH 7.4, (**b**) 0.3 mM H_2_O_2_, (**c**) pH 6.0 and (**d**) 5 mg/mL glucose.

We constructed pH [48], ROS [42] and glucose [49] multiple responses of the borate ester bonds to investigate the drug release behavior of the hydrogel in the simulated wound tissue environment of high oxidation and weak acidity. As shown in Figure 6, hydrogels composed of different molecular weights of HA (HA-free, 200 kDa-HA, and 500 kDa-HA) showed varying release profiles for EGCG. The HA-free group exhibited a rapid and high release rate, reaching over 80% after 2 days. However, the introduction of HA significantly reduced the release amount at pH 7.4, with 200 kDa-HA and 500 kDa-HA groups releasing (36.5 ± 1.8) % and (29.87 ± 1.7) %, respectively. It could be considered that in addition to ester bonds, EGCG also formed hydrogen bonds with the polymers and then was thus stably locked within the hydrogel network. Figure 6b–d show the drug release results under different response stimulation conditions. It was found that the 500 kDa-HA group had an insignificant response to H_2_O_2_, pH and glucose environment stimulation, with the release rate below 40%. However, the 200 kDa-HA group showed a gradually increasing release behavior under any stimulation and the cumulative release rate reached over 80%. Therefore, it will be used as an ideal carrier for drug controlled and sustained drug release in subsequent research.

**Figure 7:**
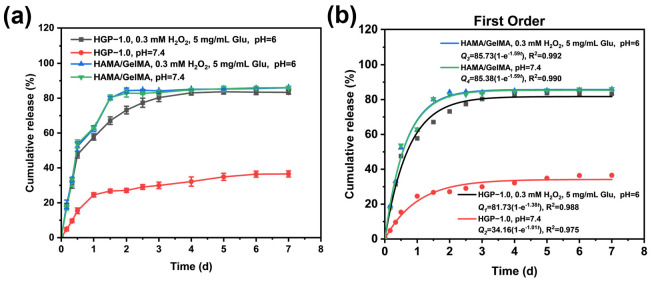
PBA-modified and non-modified EGCG-loaded hydrogels: (**a**) Release behavior (**b**) Kinetic models.

Then, low pH, high ROS and high glucose conditions were selected to investigate the responsiveness of ester bonds formed by PBA and EGCG. As shown in Figure 7a, for hydrogels HAMA/GelMA without PBA modification, a burst release behavior was observed with or without stimulation. The release reached equilibrium within 2 days, and the cumulative release rate was (83.3 ± 1.1) %. However, for the HGP-1.0 hydrogel, the drug release was only about 30% under pH 7.4. According to the results shown in Figure 5c (Degradation rate curve), it can be seen that the degradation rate after 3 days under a neutral condition is close to 50%, but EGCG in HGP-1.0 does not leak in large amounts. This indicates that most of the EGCG was still bound on GelMA by ester bonding. Under the environment of high oxidation, low pH, and high glucose stimulation, the final cumulative release rate exceeds 80%. These results demonstrate that the introduction of PBA enables stimulus-responsive release of EGCG with *o*-diol groups, facilitating controllable and sustained EGCG release from the hydrogel. Figure 7b shows the kinetic simulation of its drug release, and it was found that the drug release pattern of the EGCG-loaded hydrogel with or without modified PBA best fits the first-order reaction kinetic model with correlation coefficients R^2^ ranging from 0.975 to 0.992. It is clear that the release process in our experiments is mainly dominated by the diffusion mechanism. However, Mohsin [50] et al. investigated the drug release behavior of letrozole loaded in poly (lactic-co-glycolic acid) (PLGA) microparticles and embedded within a hybrid hydrogel. They found that the release profile best fitted the Higuchi model, with the release kinetics being predominantly governed by Fickian diffusion. Sahu [51] et al. evaluated the release kinetics of ciprofloxacin (Cipro) from polyacrylamide/dextran/carbon quantum dot (PAM/Dex/CQD) nanocomposite hydrogels. Their results demonstrated that the drug release followed the Peppas–Sahlin model, with Fickian diffusion serving as the dominant release mechanism in acidic media (other kinetic models we have tried are detailed in Supporting Information Figure S9).

**Figure 8:**
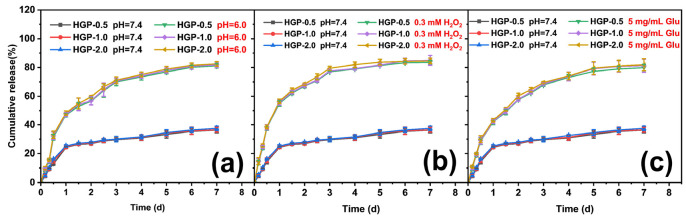
Drug release behaviors of hydrogels with different EGCG-loading ratios under various physiological conditions: (**a**) acidic environment (pH 6.0), (**b**) high oxidative stress (0.30 mM H_2_O_2_), and (**c**) hyperglycemic condition (5 mg/mL glucose).

The drug release from the 200 kDa-HA group under single stimulation conditions of pH, H_2_O_2_ and glucose was investigated, which is shown in Figure 8. It was found that the kinetic behaviors of drug release from HGP-0.5, HGP-1.0 and HGP-2.0 in the same environment were almost the same, indicating that the loading amount of EGCG did not affect the drug release behavior. Additionally, the drug release from the hydrogels in weak acid, high oxidation, and high glucose environments was found to be much greater than that under normal physiological environments. As shown in Figure 8, comparing pH 7.4 (simulating a normal physiological environment) and pH 6.0 (simulating a weakly acidic environment) under different conditions, with 300 μM H_2_O_2_ (simulating a highly oxidizing environment) or with 5 mg/mL glucose (simulating a highly glycolytic environment), a significant difference was observed in the drug release behavior and cumulative release rate. The cumulative drug release rate was less than 40% at pH 7.4 without H_2_O_2_ or glucose (normal physiological environment), whereas under any condition (such as weak acid, high oxidizing, or high glucose), the borate bond between EGCG and PBA was broken, resulting in faster drug release and a higher cumulative drug release rate (>80%). This is because the o-diol structure of glucose has a relatively high coordination efficiency with PBA. Therefore, glucose molecules will compete with EGCG for coordination with PBA, which promotes the detachment of EGCG from the borate hydrogel. Then, glucose molecules can promote the breakage of the borate bond between EGCG and PBA, resulting in the release of EGCG under the stimulus of glucose.

**Figure 9:**
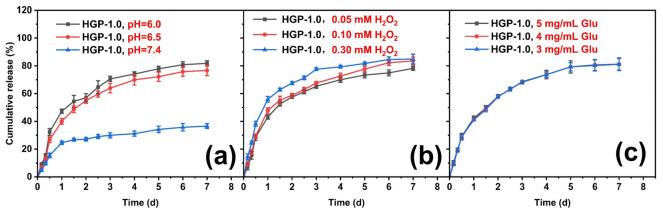
Release behavior of EGCG-loaded hydrogels under varying stimulus intensities: (**a**) different pH values, (**b**) varying H_2_O_2_ concentrations and (**c**) different glucose concentrations.

Figure 9 explores drug release at different pH, H_2_O_2_ and glucose concentrations, respectively. Within the experimental conditions of the present study, both acidic and H_2_O_2_ stimuli could result in a faster rate and more release, indicating that under either acid or H_2_O_2_ single stimulation, the borate ester bonds exhibit rapid breakage, causing EGCG bound to PBA to fall off more quickly. For glucose, it can coordinate with PBA by competing with EGCG, resulting in the release of EGCG under high glucose concentrations. As previously reported [45,52,53], a physiologic glucose concentration of 4–5 mg/mL is commonly used to model diabetic conditions. In our study, we systematically evaluated three concentrations (3, 4, and 5 mg/mL) and observed that within the concentration range of our experiment, the change in glucose concentration did not affect the release behavior.

**Figure 10:**
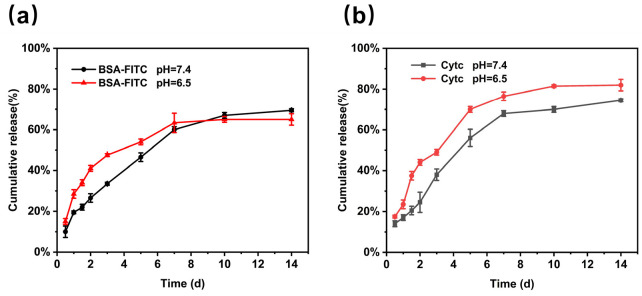
In vitro drug release from HGP hydrogels loaded with macromolecules under different conditions. (**a**) BSA-FITC; (**b**) Cytc.

In the clinic, the introduction of macromolecular drugs, such as nucleic acids and proteins, significantly enhances the wound healing process. Therefore, the release behavior of two other macromolecular drugs in hydrogels was also investigated. Figure 10 shows the release behavior of two proteins, Cytc (pI 10.8, 12–13 kDa) and BSA-FITC (pI 4.7–5.2, 66.5 kDa), in HGP hydrogels. According to the isoelectric points of BSA, it carries negative charge under neutral and weak acidic conditions, which leads to electrostatic repulsion between the BSA and the hydrogel network. As a result, BSA is mostly confined within the hydrogel by the blocking effect of network, although there may also be some hydrogen bonding between BSA and the polymers [54]. As for Cytc, which has an isoelectric point of 10.8, it primarily relies on electrostatic interactions to bind to the hydrogel network under neutral conditions. Figure 10a demonstrates that the release rate of BSA at pH 6.5 was higher than that at pH 7.4 during the initial 5 days, indicating that acidic conditions weakened hydrogen bonding and promoted drug release. However, the cumulative release at both pH values did not differ significantly, likely due to the degradation of the hydrogel itself, which contributed to the network’s disintegration and played a dominant role in later-stage release. Surprisingly, Cytc (Figure 10b) exhibited similar release kinetics to BSA despite its strong electrostatic interactions with the hydrogel. The release rate and cumulative release of Cytc were similar to those of BSA, despite the strong electrostatic interaction Cytc with the hydrogel. The release of Cytc could be attributed to the degradation of the hydrogel and the relatively small volume of Cytc, which allows it to more easily diffuse through the network. Finally, when comparing the final release rate of macromolecular proteins (~80%) in Figure 10 with that of the small-molecule EGCG (~30%) in Figure 7a, it can be found that at pH 7.4, the presence of ester bonds between EGCG and the polymer resulted in a much lower release amount, even though the hydrogel underwent serious degradation. The results of macromolecule release experiments could further confirm the importance of ester bonds.

### 2.6. Biocompatibility of Hydrogels

**Figure 11:**
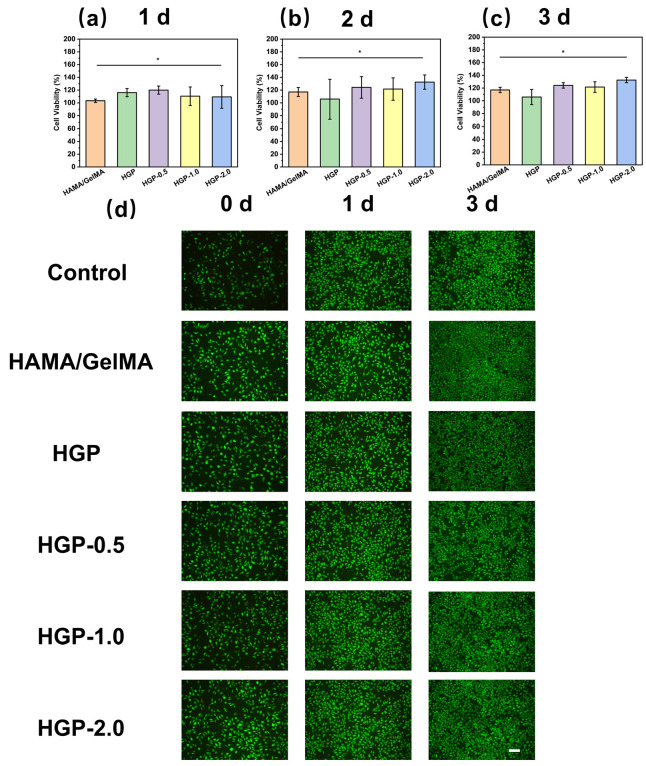
Cytocompatibility of hydrogels. Cell survival in co-culture with different concentrations of hydrogel extracts according to the CCK-8 assay results for (**a**) 1 day, (**b**) 2 days and (**c**) 3 days, * p < 0.05, *n* = 5. (**d**) Inverted fluorescence images of cells cultured with various hydrogel extracts after different times. Scale bars: 200 μm, *n* = 3.

The cytocompatibility of the hydrogels was evaluated using the CCK-8 assay and LIVE/DEAD staining. As shown in Figure 11a–c, L929 cells were co-cultured with extracts of various hydrogels at concentration of 100 mg/mL for 1, 2, and 3 days, and the cell viability remained above 95% in all cases. Figure 11d presents the results of L929 cells incubated with 100 mg/mL extract, where Calcein-AM/PI staining was used to label live and dead cells, respectively. The results demonstrated a significant proliferation trend across all samples, with the drug-loaded hydrogel group exhibiting a more pronounced proliferation trend compared to the blank control group. These findings indicate that the prepared hydrogels possess excellent cytocompatibility.

**Figure 12:**
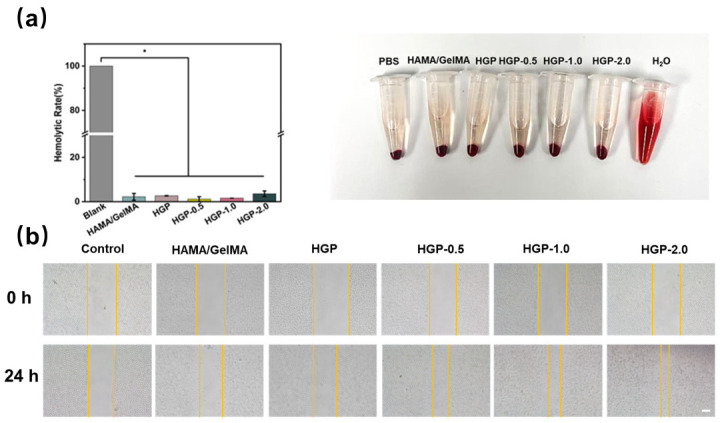
Hemolysis and scratch assay. (**a**) Hemocompatibility of the hydrogels, * *p* < 0.05, n = 5. (**b**) Cell migration behavior estimated by in vitro scratch assay. Scale bar = 100 μm, n = 3.

In addition, hemolysis studies were conducted using rat red blood cells (RBCs) to assess the hemocompatibility of the hydrogel extracts at a concentration of 100 mg/mL. As shown in Figure 12a, all hydrogels had a hemolysis rate of less than 5%, indicating excellent hemocompatibility. Furthermore, an in vitro scratch assay was performed to evaluate the effects of the hydrogel extracts on cell migration. We performed in vitro scratch wound healing experiments to investigate the potential of EGCG-loaded hydrogels to promote L929 fibroblast migration. As shown in Figure 12b, the HAMA/GelMA hydrogel and HGP hydrogel without EGCG were almost unable to promote scratch healing. With increasing drug loading ratios, the wound healing rate increased. Figure S11 shows the quantification of cell migration rates, which increased from 42.9% for HGP-0.5 to 62.1% for HGP-2.0. The EGCG present in the extract enhances cell migration, suggesting that the hydrogel may enhance tissue regeneration and accelerate wound healing. Overall, these results confirm that the hydrogels are biocompatible and capable of promoting cell migration and proliferation.

### 2.7. Antioxidant Efficiency of Hydrogels

**Figure 13:**
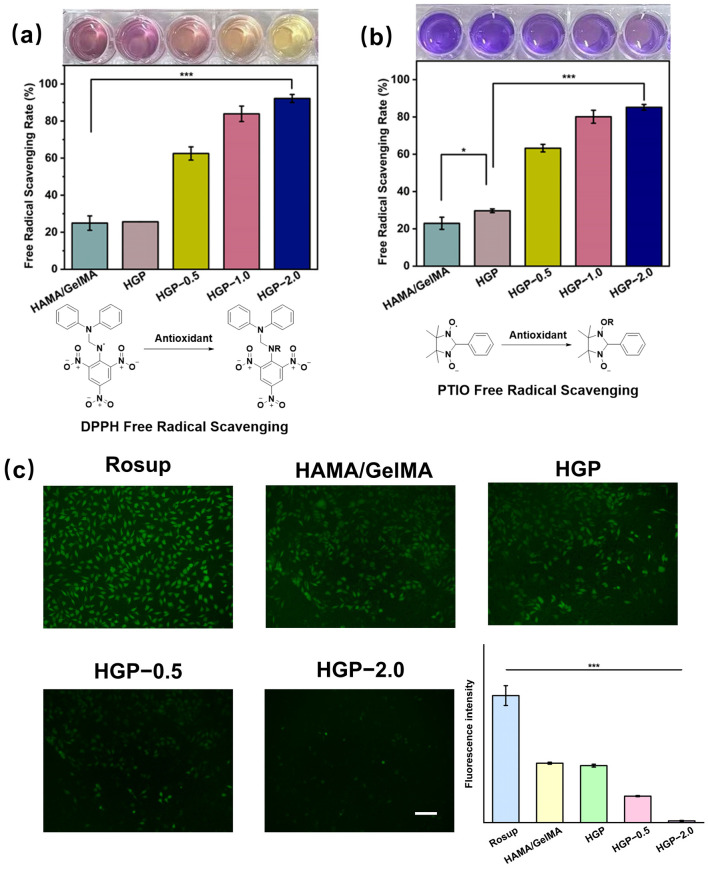
Free radical scavenging and antioxidant properties of hydrogels. Free radical scavenging efficiency and mechanisms of hydrogel on (**a**) DPPH and (**b**) PTIO free radical, * *p* < 0.05, *** *p* < 0.001, *n* = 5. (**c**) DCFH-DA staining assay to characterize the intracellular ROS scavenging ability of hydrogels and their quantized results from the Image J. Scale bars: 200 μm. *** *p* < 0.001, *n* = 5.

The antioxidant properties of the hydrogels were investigated through DPPH (nitrogen radical) and PTIO (oxygen radical) scavenging assays. As shown in Figure 13, the HGP-0.5 hydrogel exhibited a radical scavenging efficiency of approximately 60% for both DPPH and PTIO, which was significantly higher than that of the drug-free group (~25%). This demonstrates that the incorporation of EGCG effectively scavenged nitrogen and oxygen radicals, and the scavenging efficiency increased with the drug loading concentration. Specifically, the radical scavenging efficiencies of HGP-1.0 and HGP-2.0 were 80% and 85%, respectively, reaching high levels of antioxidant activity.

In addition, intracellular ROS scavenging ability was assessed by DCFH-DA staining after treating L929 cells with H_2_O_2_ to induce oxidative stress. As shown in Figure 13c, the positive control group (treated with H_2_O_2_) exhibited high green fluorescence intensity, indicating the successful induction of ROS inside the cells. However, in the drug-loaded groups, the green fluorescence intensity significantly decreased with increasing EGCG concentration. The HGP-2.0 group exhibited the lowest fluorescence intensity, indicating the best antioxidant effect.

## 3. Conclusions

In this study, a dynamically crosslinked hydrogel integrating PBA and EGCG was successfully synthesized, showcasing remarkable mechanical properties, injectability, biodegradability, and multi-stimuli responsiveness. The introduction of PBA groups significantly augmented the mechanical strength of the hydrogel, whereas the dynamic boronate ester bonds conferred responsiveness to ROS, pH, and elevated glucose concentrations. Rheological and mechanical characterizations were conducted to elucidate the impact of PBA and EGCG on the network structure and performance of the hydrogel. Regarding drug delivery applications, the hydrogel manifested exceptional sustained and controlled release capabilities for both small-molecule drugs and macromolecular therapeutics. It achieved stimulus-triggered release behaviors under conditions of high ROS levels, low pH, and hyperglycemia as well as accurate/sustained EGCG release only when needed, which improves the utilization of EGCG. Cytocompatibility evaluations verified the hydrogel’s outstanding biocompatibility, and antioxidant investigations confirmed its proficiency in efficiently scavenging intracellular ROS. In essence, this multifunctional hydrogel could present a promising platform for drug delivery and tissue engineering applications. These results offer valuable perspectives for the design of advanced hydrogel systems intended to alleviate oxidative stress and facilitate tissue regeneration.

## 4. Materials and Methods

### 4.1. Reagents and Materials

Gelatin (Type A), Alizarin Red S (ARS), 2-Phenyl-4,4,5,5-tetramethylimidazoline-1-oxo, 3-oxide (PTIO, 98%) and 1,1-diphenyl-2-picrylhydrazine (DPPH, 98%) were purchased from Shanghai Yuanye Biotech Co., Ltd. (Shanghai, China). Hyaluronic acid (HA, 200 kDa/500 kDa) was obtained from Shanghai Canspec. N-Hydroxy succinimide (NHS), 1-(3-Dimethylaminopropyl)-3-ethylcarbodiimide (EDC, 98%) and methacrylic anhydride (MA, >94%) and photo initiator 2-Hydroxy-4′-(2-hydroxyethoxy)-2-methylpropiophenone (I2959, >98%) were purchased from Shanghai Titan Technology Co., Ltd. (Shanghai, China). Cytochrome c (Cytc, >95%), 3-aminobenzeneboronic acid (APBA, >98%) was purchased from Shanghai Aladdin Biochemical Technology Co., Ltd. (Shanghai, China). Epigallocatechin gallate (EGCG, 95%) was purchased from Shanghai Meryer Biotech Co. Ltd. (Shanghai, China). Cell counting kit-8 (CCK-8) was purchased from Beyotime Biotechnology (Shanghai, China). Bovine serum albumin-FITC (BSA-FITC, >95%), Calcein/PI Cell Viability/Cytotoxicity Assay Kit and Reactive Oxygen Species Assay Kit (DCFH-DA) were purchased from Dalian Meilun Biotechnology Co., Ltd. (Dalian, China).

### 4.2. Synthesis and Characterization of Methacrylated Hyaluronic Acid (HAMA)

Briefly, HA (1.6 g, 4 mmol) was added to a mixture of 100 mL ultrapure water and *N, N*-dimethylformamide (volume ratio 3:1). The pH was adjusted to 9, and then 1.5 equivalents of MA (0.9 mL, 6 mmol) were slowly added while stirring at 4 °C. The reaction proceeded for 6 h, and then stirred overnight at 4 °C. The product was purified by dialysis using deionized water through dialysis membranes (MWCO 8000–14,000 Da). The structure and degree of substitution (DS) of the product were determined by ^1^H NMR (Bruker AVANCE 400 MHz, Billerica, Switzerland) and Fourier Transform Infrared (FTIR, Nicolet 6700, Nickelodeon, Madison, Wisconsin, USA) spectroscopy.

### 4.3. Synthesis and Characterization of Phenylmethacrylated Gelatin (GelMA-PBA)

The synthesis of GelMA followed a similar protocol described in previous studies [36]. Briefly, 10 g of gelatin was dissolved in 0.25 mol/L CBS (Na_2_CO_3_/NaHCO_3_) buffer solution at 35 °C. Then, 1 mL of MA was added, and the reaction proceeded for 4 h. The product was dialyzed in deionized water (MWCO 8000–14,000 Da) for 3 days. To prepare GelMA-PBA, 1 g of GelMA was dissolved in a mixture of water and N, N-dimethylformamide (5:1). The reaction was carried out under a nitrogen atmosphere. Then, the EDC (383.4 mg, 2 mmol) and NHS (230.18 mg, 2 mmol) were added and reacted for 30 min. After pH was adjusted to 6–6.5 and of APBA (256.6 mg, 2 mmol) was added for a 24 h reaction. The product was purified by dialysis using ultrapure water (MWCO 8000–14,000 Da) for 5 days. The structure and degree of substitution (DS) were determined by ^1^H NMR and FTIR.

### 4.4. Preparation of HAMA/GelMA and HGP Hydrogels

According to Table 1, different concentrations of EGCG were dissolved in 2‰ I2959 aqueous solutions, and then 1% HAMA and 10% GelMA or GelMA-PBA were added. The mixture was stirred at 35 °C overnight. The pre-gel solution was transferred to molds, and UV light was applied for 60–120 s (365 nm, 40 mW/cm^2^). Hydrogels formed by HAMA and GelMA or GelMA-PBA are referred to as HAMA/GelMA and HGP hydrogels, respectively. The EGCG loadings of 0.05%, 0.1%, and 0.2% were used for the corresponding hydrogels (HGP-0.5, HGP-1.0, HGP-2.0). The hydrogel extracts were obtained by immersing freshly prepared hydrogels in PBS buffer (pH = 7.4, 10 mM) or cell culture medium for 24 h. The grafting success of PBA was determined using the ARS staining method, which involved mixing 100 mg the hydrogel with 0.1% ARS solution and measuring the UV spectrum of the resulting solution.

### 4.5. Physical Properties of HAMA/GelMA and HGP Hydrogels

The microstructure of the freeze-dried hydrogels was characterized using scanning electron microscopy (SEM, S-3400N, HITACHI, Hitachinaka, Japan). Measurement of swelling and degradation rates of hydrogels similar to that shown in the previous report [37,38]. The swelling ratio (SR) of the hydrogels was investigated by immersing the freeze-dried hydrogels in PBS buffer (pH = 7.4, 10 mM) at 37 °C and weighing the hydrogel at fixed intervals until its weight became constant. The degradation rate (DR) of the hydrogels was determined by immersing freshly prepared hydrogels in PBS buffer solution and weighing the hydrogels after fixed time intervals.

The water vapor transmission rate (WVTR) of the hydrogel was determined by measuring the amount of water vapor passing through the hydrogel in a 24 h sealed and dry environment, calculated using Formula (1):

WVTR *=* [*(m_a_* − *m_b_)/S*] × 100% (1)

where *m_a_* is the initial mass of water in the container, *m_b_* is the final mass, and *S* is the area of the container opening.

The swelling behavior of the hydrogels was investigated by immersing the freeze-dried hydrogels in PBS (pH = 7.4, 10 mM) buffer solution at 37 °C, weighing the hydrogel at fixed intervals until its weight became constant (*t* = 2, 4, 6, 8, 10, 12, 24 h). The swelling ratio (SR) was calculated using Formula (2):

SR = [(*m* − *m_0_*)/*m_0_*] × 100% (2)

where *m* is the mass of the swollen hydrogel and *m*_0_ is the mass of the freeze-dried hydrogel.

### 4.6. Mechanical Properties of Hydrogels

The mechanical properties of the hydrogels (d = 15 mm, h = 8 mm) were evaluated using a dynamic mechanical testing system (ElectroForce 3200, TA Instruments, Eden Prairie, Minnesota, USA) at a constant strain rate of 1 mm/min. Young’s modulus of the hydrogels was calculated from the slope of the linear region of the stress–strain curve (0–10% strain), with testing performed at room temperature (25 °C, 60% humidity).

### 4.7. Rheological Properties of Hydrogels

The rheological properties of the hydrogels were evaluated using a rotational rheometer (MCR 302, Anton Paar, Graz, Austria). Hydrogels (h = 1 mm, d = 45 mm) were placed between parallel plates with a diameter of 50 mm and a gap of 1 mm. The storage modulus (G′) and loss modulus (G″) were measured, with a fixed angular frequency of 10 rad/s at 37 °C. Strain amplitude sweep tests (γ = 0.01–100%) were performed to detect the critical strain point. Additionally, the modulus during 5 cycles of low (γ = 1%) and high (γ = 100%) strain switching was measured. Finally, the viscosity of the gels was measured across a shear rate range of 1–1000 s^−1^.

### 4.8. In Vitro Drug Release Behavior of Hydrogels

First, 500 mg of hydrogel was placed in 10 mL of PBS (with different pH, H_2_O_2_, and glucose concentrations) and incubated in a constant temperature shaker (37 °C, 100 rpm). At each time point, the supernatant was taken and replenished with the same volume of PBS. Since EGCG is easily oxidized and its UV absorbance increases, the release of EGCG was quantified using the Folin–Ciocalteu method. A 20 μL sample of release solution was mixed with 100 μL of Folin–Ciocalteu reagent (10% *v*/*v*) in a 96-well plate. After adding 80 μL of sodium carbonate solution (7.5% *w*/*v*), the solution was incubated in the dark for 30 min, and the absorbance at 765 nm was measured using a microplate reader (xMark, Bio-RAD, Hercules, California, USA).

To study the release of macromolecular drugs, two model proteins, Cytc and BSA-FITC, with different isoelectric points and molecular weights, were selected. The release of Cytc was determined by measuring the UV absorbance at 408.5 nm, while BSA-FITC was quantified by fluorescence intensity at 520 nm. The cumulative release rate of drugs was calculated using Formula (3):

(3) $$\;Cumulative\;release\;(\%)=\frac{C_{n}\;\times \;V_{n}+\sum C_{n-1}\times \;V}{m}\times 100\%$$

where *C_n_* is the concentration of drug in PBS buffer at a given moment (mg/mL). *V_n_* is the volume of PBS buffer (mL), and *V* is the volume of buffer removed each time for testing. ∑*C_n_*_−1_ is the cumulative concentration (mg/mL) of the drug in the first *n* − 1 times of PBS. *m* is the total weight of drug loaded in the hydrogel (mg).

To deeply explore the release mechanism, the release behavior of hydrogels with and without modified PBA under different conditions was fitted with kinetic models. The relationship between drug release amount and time in the first-order kinetic model is shown in Equation (4):

(4) $$Q_{\infty }=Q\;(1-\frac{a}{Q_{\infty }}e^{-k_{1}t})$$

Here, *Q*_∞_ represents the maximum drug release amount, *Q_t_* is the cumulative release amount at time *t*, a is a constant term, and *k*_1_ is the first-order reaction rate constant. The release rate is proportional to the remaining drug concentration.

### 4.9. Cell Culture

L929 mouse fibroblast cells (from Dalian Meilun Biotechnology Co., Ltd. (Dalian, China), CL0738) were cultured and passaged in a cell incubator (37 °C, 5% CO_2_) for at least three passages, within 10 generations. All reagents used in cell experiments were sterilized by autoclaving or filtration through a 0.22 μm membrane.

### 4.10. Cell Compatibility and Hemolysis Tests

Cell compatibility of the hydrogels was evaluated using the CCK-8 assay and Calcein-AM/PI staining method. Different types of hydrogel extracts (100 mg/mL) were co-cultured with L929 cells (1.0 × 10^4^ cells/well) for 1 day, 2 days and 3 days. The culture medium was replaced with serum-free medium containing 10% CCK-8. Absorbance at 450 nm was measured using a microplate reader. For LIVE/DEAD staining, cells were incubated with Calcein-AM/PI dye for 30 min and observed under an inverted fluorescence microscope (EVG, Nikon, Melville, New York, USA). Distilled water and PBS were used as positive and negative controls for hemolysis tests on rat red blood cells (RBCs). The hemolysis rate was determined by measuring absorbance at 542 nm, as shown in Formula (5):

(5) $$\;Hemolysis\;Rate\;(\%)=\frac{\mathrm{OD}\;\left(\mathrm{sample}\right)\;-\;\mathrm{OD}\;\left(\mathrm{PBS}\right)}{\mathrm{OD}\;(H_{2}O)\;-\;\mathrm{OD}\;\left(\mathrm{PBS}\right)}\times 100\%$$

where *OD* (sample) represents the absorbance of the sample group, *OD* (H_2_O) denotes the absorbance of H_2_O as the positive control, and *OD* (PBS) indicates the absorbance of PBS as the negative control.

### 4.11. In Vitro Scratch Healing Assay

Hydrogel extracts were prepared by soaking the hydrogels in serum-free medium. L929 cells (1.0 × 10^6^ cells/well) were seeded into 6-well plates and incubated in serum-free medium for 4 h. A scratch was created in the cell monolayer using a 200 μL pipette tip. After washing with PBS to remove floating cells and debris, the cells were treated with various hydrogel extracts. Cell migration was photographed before and after incubation, and the migration rate was obtained by Image J2 (Fiji) software.

### 4.12. Free Radical Scavenging Ability of Hydrogels

The free radical scavenging ability of the hydrogel was assessed by scavenging assays using two stably present (normal physiological environment) free radicals, DPPH and PTIO. First, 100 mg of hydrogel was directly added to 1 mL of 100 μM DPPH ethanol solution and incubated for 30 min. After centrifugation using a centrifuge (3000 rpm, 3 min), the absorbance of the supernatant at 519 nm was measured using a microplate reader. For PTIO, 100 mg of hydrogel was added to 1 mL of 0.5 mmol PTIO solution, incubated for 5 h, centrifuged at 3000 rpm for 3 min and the absorbance of the supernatant was measured at 557 nm. Clearance efficiency was calculated using Equation (6):

(6) $$\mathrm{Free}\;\mathrm{Radical}\;\mathrm{Scavenging}\;\mathrm{Rate}\;\left(\%\right)=[1\;-\;\frac{\mathrm{OD}\;\left(\mathrm{sample}\right)\;-\;\mathrm{OD}\left(\mathrm{control}\right)}{\mathrm{OD}\left(\mathrm{free}\;\mathrm{radical}\right)}]\times 100\%$$

where *OD* (control) is the absorbance of the sample group, *OD* (sample) is the absorbance of the sample group treated the free radical solution and *OD* (free radical) is the absorbance of the free radical solution. 

### 4.13. Intracellular Antioxidant Ability Assay

The oxidative model was created by treating cells with 1 mM H_2_O_2_. ROS scavenging capacity was assessed using the DCFH-DA kit. L929 cells were co-incubated with 1 mM H_2_O_2_ and hydrogel extract (100 mg/mL) for 4 h, and then stained with DCFH-DA solution (10 μM) for 15 min. The oxidative degree of the cells was observed under an inverted fluorescence microscope.

### 4.14. Statistical Analysis

Data were plotted using OriginPro 2025 (10.2.0.188) software (Origin Lab, Northampton, Massachusetts, USA). All data are expressed as mean ± standard deviation (s. d.), and two-way ANOVA was used for comparison. Tukey’s post hoc test was used for statistical analysis of differences between two groups. Statistical significance was set at * *p* < 0.05, ** *p* < 0.01, *** *p* < 0.001.

## Figures and Tables

**Table 1:** The composition of hydrogels.

Name	HAMA (mg/mL)	GelMA (mg/mL)	GelMA-PBA (mg/mL)	EGCG (mg/mL)
HAMA/GelMA	10	100	0	0
HGP	10	0	100	0
HGP-0.5	10	0	100	0.5
HGP-1.0	10	0	100	1
HGP-2.0	10	0	100	2

## Data Availability

Data are contained within the article and Supplementary Materials.

## Supplementary Materials

The following supporting information can be downloaded at https://www.mdpi.com/article/10.3390/gels11050370/s1: Figure S1. FTIR spectra of HA and HAMA; Figure S2. FTIR spectra of gelation, GelMA and GelMA-PBA; Figure S3. Schematic diagram of the cross-linking mechanism of EGCG with gelatin and hyaluronic acid; Figure S4. Water vapor transmission rate (WVTR) of various hydrogels; Figures S5–S8. Standard curve of EGCG under various condition; Figure S9. Modeling of drug release kinetics from EGCG-loaded hydrogels with and without modified PBA; Figure S10. Standard curves of BSA and Cytc at different pHs; Figure S11. Quantification of the extent of cell migration; Movie S1. Characterization of hydrogel injectability.

## Abbreviations

The following abbreviations are used in this manuscript:

HA	Hyaluronic acid
PBA	Phenylboronic acid
MA	Methacrylic anhydride
ARS	Alizarin Red S
EGCG	Epigallocatechin gallate
EDC	1-(3-Dimethylaminopropyl)-3-ethylcarbodiimide
NHS	N-Hydroxy succinimide
DPPH	1,1-Diphenyl-2-picrylhydrazyl radical 2,2-Diphenyl-1-(2,4,6-trinitrophenyl) hydrazyl
PTIO	2-Phenyl-4,4,5,5-tetramethylimidazoline-3-oxide-1-oxyl
DS	Degree of substitution
WVTR	Water vapor transmission rate
OD	Optical density
Cytc	Cytochrome c
SR	Swelling rate
DR	Degradation rate
