# Supplementary material for: Dynamic Boronate Ester Based Hydrogel with Enhanced Mechanical Properties and Multi-Stimuli-Triggered Release for Tissue Repair and Antioxidant Therapy
Source: Gels. 2025 May 18;11(5):370. doi: 10.3390/gels11050370 (PMC12111388; doi:10.3390/gels11050370)
Supplement: Supplementary file 1 [file gels-11-00370-s001.zip › gels-3605026-supplementary.pdf]

Supporting Information

Dynamic Boronate Ester Based Hydrogel with Enhanced Mechanical Properties and Multi-Stimuli-Triggered Release for Tissue Repair and Antioxidant Therapy

Fangyi Liu [1], Gaoyang Li [1], Zhenhui An [1], Sijia Wang [2*], Shouhong Xu [1*], Honglai Liu [1]

1 Key Laboratory for Advanced Materials, School of Chemistry and Molecular Engineering, East China University of Science and Technology, Shanghai 200237, China

2 College of Pharmacy, Henan University of Chinese Medicine, Zhengzhou 450046, China

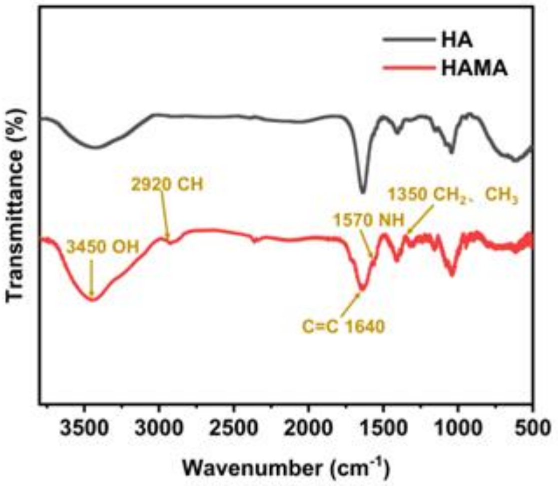

Figure. S1 FTIR Spectra of HA and HAMA.

The FTIR spectrum exhibits a broad peak at approximately 3450 cm^{-1}, corresponding to the O-H stretching vibration of hydroxyl groups. The absorption band around 2920 cm^{-1} is attributed to the C-H stretching vibration of alkyl groups. A distinct peak near 1700 cm^{-1} is associated with the C=O stretching vibration, characteristic of ester or carboxyl functional groups. The peak observed at approximately 1640 cm^{-1} corresponds to the C=C stretching vibration, indicative of acrylate groups. Additionally, the band around 1570 cm^{-1} is ascribed to the N-H bending vibration of amide II.

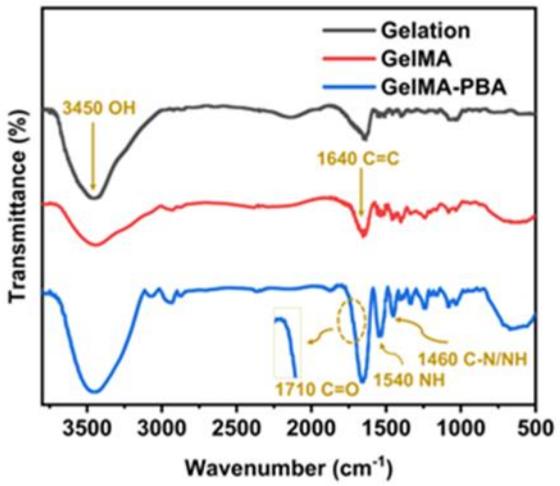

Figure. S2 FTIR Spectra of Gelation, GelMA and GelMA-PBA.

The FTIR spectrum exhibits a broad absorption band at 3450 cm⁻¹, corresponding to the N-H and O-H stretching vibrations, characteristic of amide and hydroxyl functional groups. The peak at 1710 cm⁻¹ is attributed to the C=O stretching vibration, indicative of ester groups. The absorption band at 1640 cm⁻¹ corresponds to the C=C stretching vibration, associated with methacryloyl groups. Additionally, a peak around 1600 cm⁻¹ is assigned to the C=C stretching vibration of the benzene ring, originating from phenylboronic acid, while the band at 1540 cm⁻¹ corresponds to the N-H single-bond stretching vibration. The absorption at 1460 cm⁻¹ is attributed to either C-N or N-H stretching vibrations. Finally, the peak at 1330 cm⁻¹ is ascribed to the B-O stretching vibration, serving as a characteristic marker of phenylboronic acid.

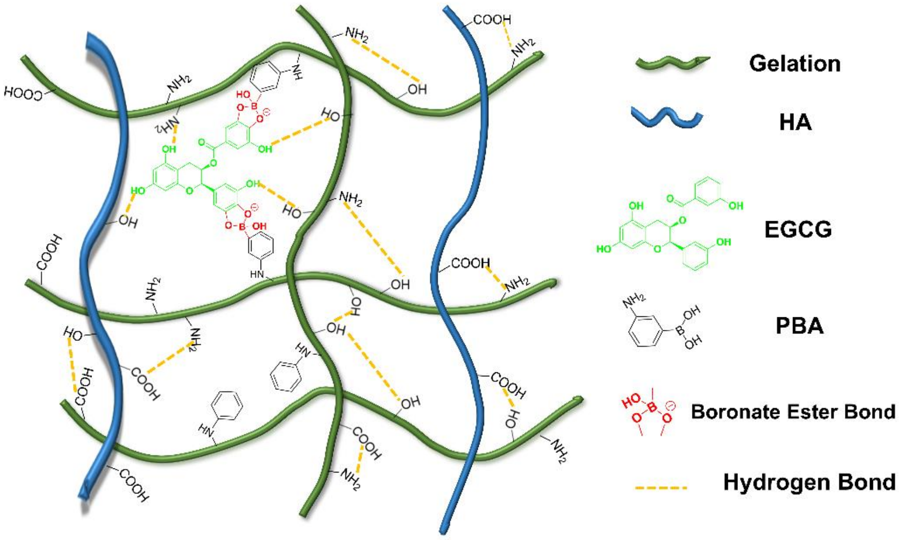

Figure. S3 Schematic diagram of the cross-linking mechanism of EGCG with gelatin and hyaluronic acid.

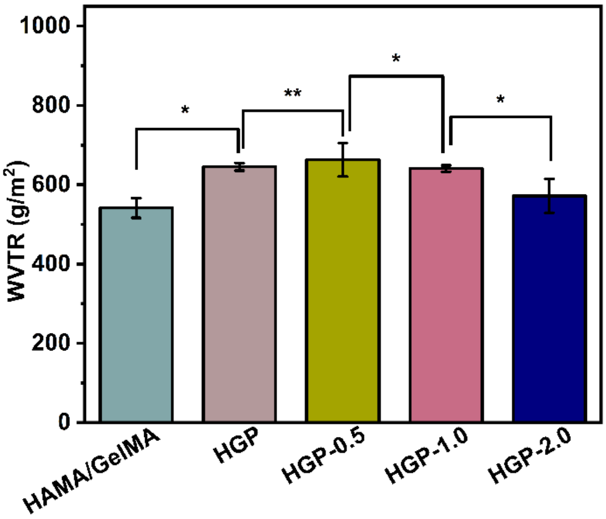

Figure. S4 Water vapour transmission rate (WVTR) of various hydrogels.

Referring to previous reports in the literature, a modified ASTM standard method (ASTM E96-90) was used to determine the water vapour transmission rate of various hydrogels over a 24 h period in order to examine their permeability.

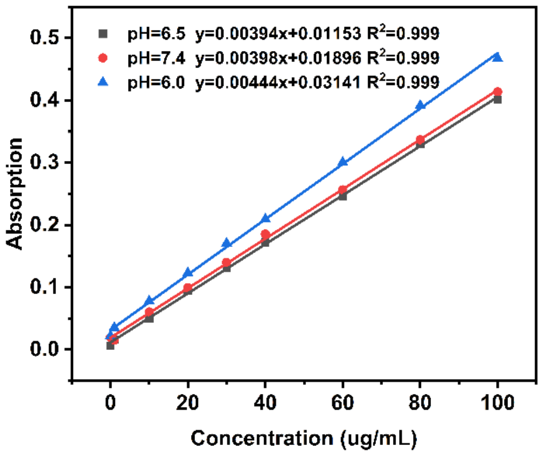

Figure. S5 Standard curve of EGCG for different pH (pH=6.0, 6.5, 7.4).

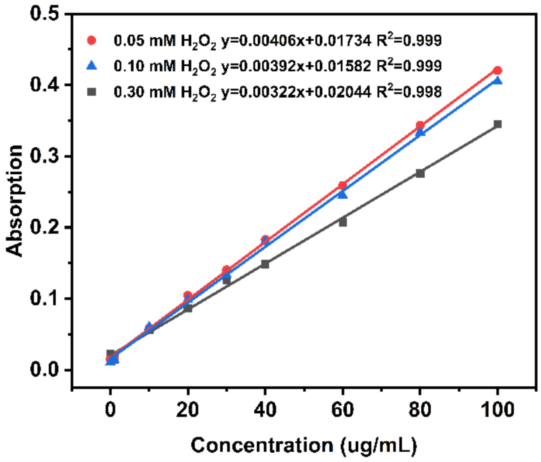

Figure. S6 Standard curves of EGCG for different H_2O_2 concentrations (0.05 mM, 0.10 mM, 0.30 mM).

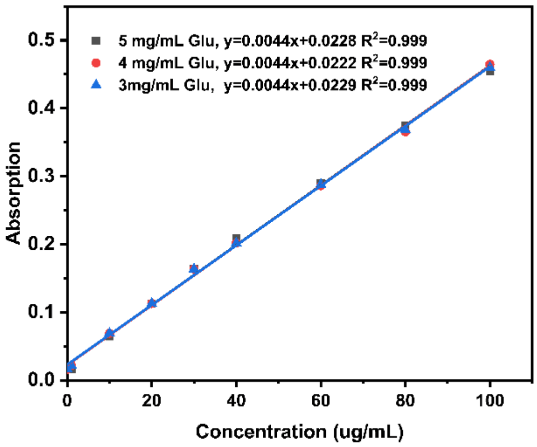

Figure. S7 Standard curves of EGCG with different glucose concentrations (3 mg/mL, 4 mg/mL, 5 mg/mL).

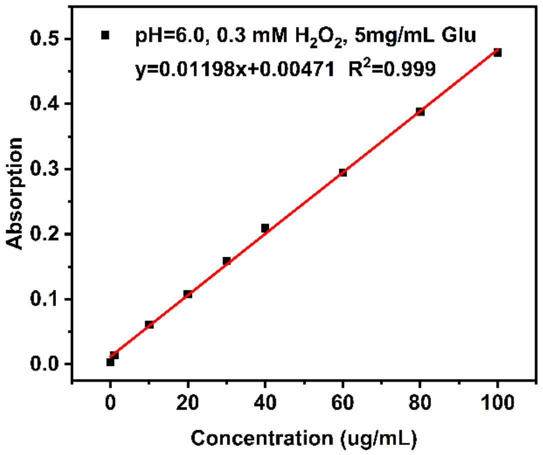

Figure. S8 Standard curve of EGCG at pH=6.0, glucose concentration of 5 mg/mL and H_2O_2 concentration of 0.30 mM.

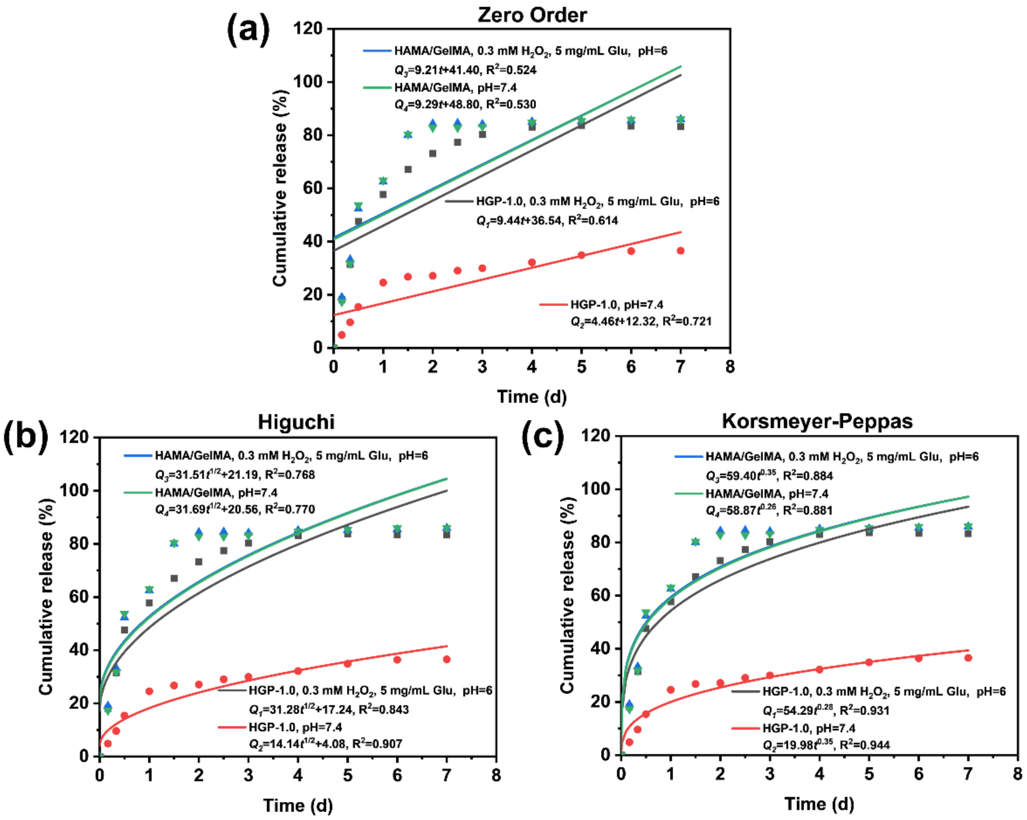

Figure. S9 Modeling of drug release kinetics from EGCG-loaded hydrogels with and without modified PBA, (a) zero-level model, (b) Higuchi model, (c) Korsmeyer-Peppas model.

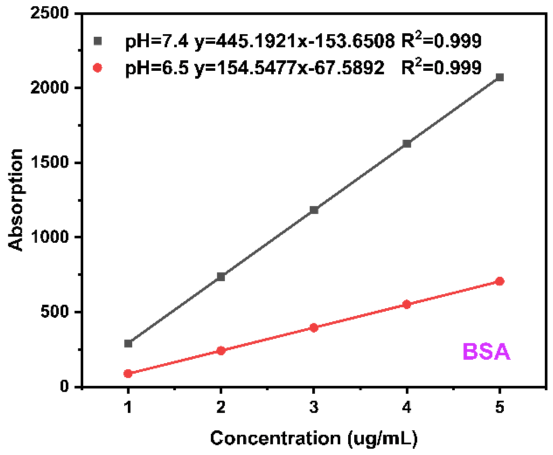

Figure. S10 Standard curves of BSA and Cytc at different pHs.

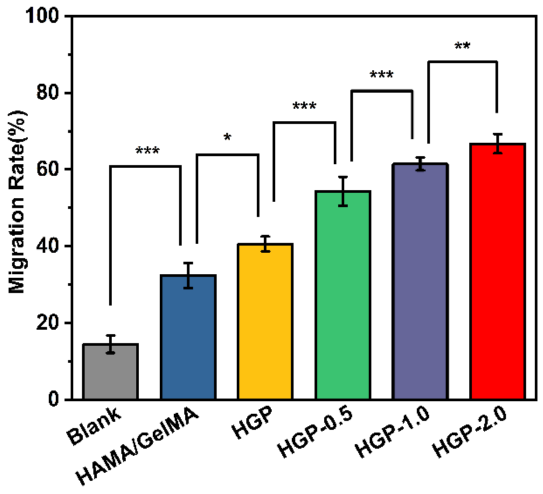

Figure. S11 Quantification of the extent of cell migration.
